# Regressive Neuroblastoma: Widely Accepted, Underreported Histology

**DOI:** 10.1002/ccr3.72640

**Published:** 2026-04-29

**Authors:** Tom Membrez, Jim C. H. Wilde, Oliver Sanchez, Danai Papangelopoulou, Fabienne Gumy‐Pause, Anne‐Laure Rougemont

**Affiliations:** ^1^ Pediatric and Fetoplacental Pathology Unit, Division of Clinical Pathology, Diagnostic Department Geneva University Hospitals and Faculty of Medicine, University of Geneva Geneva Switzerland; ^2^ Division of Pediatric Surgery, Department of Women, Child and Adolescent Geneva University Hospitals and Faculty of Medicine, University of Geneva Geneva Switzerland; ^3^ Division of Pediatric Oncology and Hematology, Department of Women, Child and Adolescent Geneva University Hospitals and Faculty of Medicine, University of Geneva Geneva Switzerland

**Keywords:** differentiation, fibrosis, maturation, mature ganglion cells, regressed neuroblastoma, spontaneous regression

## Abstract

Neuroblastoma accounts for approximately 8% of all pediatric cancers and up to 15% of pediatric cancer‐related deaths in malignant cases; neuroblastoma is the most common malignancy in infancy. Notably, up to 50% of tumors may undergo spontaneous regression without treatment. However, detailed histological descriptions of regressed neuroblastoma remain limited in the literature. We report the histological features of a particularly illustrative case, characterized by edema, mild collagen deposition, evidence of prior hemorrhage, and calcifications. Cellularity was minimal, consisting of rare, scattered mature‐appearing ganglion cells, without neuropil or neuroblastic clusters. Accurate recognition of these morphological features in surgical specimens is essential for optimal treatment planning.

## Key Clinical Message

Localized neonatal neuroblastoma may exhibit spontaneous histological regression indistinguishable from post‐chemotherapy changes. This report fills a gap in the literature and provides a reference framework for pathologists diagnosing spontaneous regression.

## Graphical abstract

Whole‐mount section of the regressed neuroblastoma within the adrenal gland, showing a loose, collagenous, and edematous stroma.

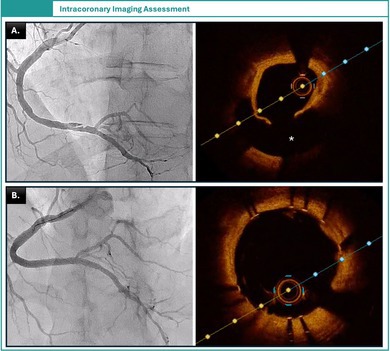

## 1 Introduction

Neuroblastoma is a tumor originating from the neural crest progenitor cells of the sympathetic nervous system [1] and is reported as the most common extracranial solid tumor of childhood [2]. Most of the cases occur in patients under 5 years of age with a mean diagnostic age of 2 years [3]. It represents 8% of all pediatric cancers and, when malignant, accounts for up to 15% of pediatric cancer deaths. The heterogeneous behavior of this tumor poses a major challenge for clinical management, as it may either differentiate and mature into a benign ganglioneuroma or, alternatively, manifest as a highly malignant and aggressive neoplasm with metastatic potential and resistance to multimodal therapy [4]. Age, stage, and biological/molecular parameters influence prognosis. Perinatal neuroblastoma most often presents as a localized tumor (approximately 90% of cases), typically arising in the adrenal gland. They are usually low stage (stage L1) according to the International Neuroblastoma Risk Group Staging System [5], and only rarely exhibit adverse molecular features, such as *MYCN* gene amplification [6]. Localized neuroblastoma in infants carries an excellent prognosis, with a notably high rate of spontaneous regression, occurring in approximately 50% in the absence of treatment [7, 8, 9].

Several hypotheses have been proposed regarding the cellular and molecular mechanisms underlying this phenomenon. Although spontaneous regression is frequent and clinically important in neonatal localized neuroblastoma, there remains a notable lack of literature providing clear documentation, high‐quality images, and detailed explanations on how to recognize patterns of regression through both macroscopic and microscopic evaluation during the diagnostic process. Histology remains poorly and incompletely documented, with available descriptions largely confined to older black‐and‐white reports.

Recognizing the morphological characteristics of regressed tumors in surgical specimens is crucial for patient management, as it offers valuable prognostic insights that directly guide subsequent treatment decisions.

## 2 Case History

A one‐month‐old girl, born after a full‐term uneventful pregnancy and with no significant medical history, underwent abdominal ultrasound evaluation for preauricular pits and suspected congenital hip dislocation, which incidentally revealed a homogeneous, hyperechogenic mass in the right adrenal gland. Urinary catecholamine levels were mildly elevated for age, and MIBG scintigraphy demonstrated increased metabolic activity within the mass. Neonatal neuroblastoma was diagnosed, and staging revealed no distant metastasis.

## 3 Investigations, Treatment and Final Diagnosis

Monthly ultrasound follow‐up was initiated in accordance with the SIOPEN Low and Intermediate Risk Neuroblastoma European Study (LINES). Urinary catecholamine levels normalized within 1 month of diagnosis. Over a 15‐month period, ultrasound follow‐up showed spontaneous reduction in mass volume, decreasing in size from 3.6 × 3.2 × 3.8 cm to 1.9 × 1 × 1.5 cm. At 48 weeks, MRI revealed a persistent cystic mass with small foci of calcification. Retroperitoneoscopic resection was performed at 15 months of age following the LINES protocol, without prior chemotherapy.

### 3.1 Pathology Findings

At macroscopic examination, the lesion consisted of a well‐demarcated nodule with a smooth white, shiny surface and an enucleated appearance. On section, the lesion had a homogeneous appearance, myxoid‐like, and translucent. The pre‐existing adrenal gland was observed at the periphery.

Histology revealed a relatively well‐demarcated nodular lesion, largely replacing the adrenal gland medulla. The lesion was very sparsely cellular showing a loose and edematous stroma, with some collagen bundles and numerous vessels. Iron‐laden macrophages, indicative of previous hemorrhage, and calcifications were also seen. Rare ganglion cells with a mature appearance were scattered throughout the lesion; there were only exceptional isolated slightly immature binucleate ganglion cells, but no neuropil nor neuroblastic cell clusters. On immunohistochemical examination, the rare ganglion cells expressed S100 protein and showed PHOX2B nuclear positivity.

**FIGURE 1:**
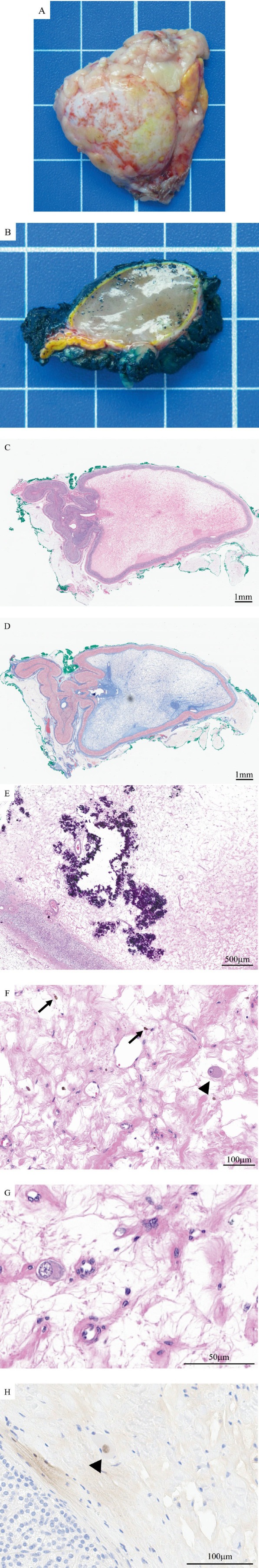
Gross findings. The grid scale is 1 × 1 cm. (A) Adrenalectomy specimen with a well‐defined nodular lesion showing a white surface and petechial hemorrhage. (B) On cut section, the lesion shows a homogenous and translucent aspect; preserved adrenal tissue is seen at the periphery (lower left). Histological findings. (C and D) Whole‐mount view of the lesion and its relationship with the normal adrenal gland parenchyma at the periphery (H&E stain and Masson's trichrome), showing a loose, collagenous, and edematous stroma. (E) Foci of calcification are seen (H&E). (F and G) The stroma is loose, mainly edematous, with sparse collagen bundles, variable thin‐walled vessels, few iron‐laden macrophages (arrows), and rare mature ganglion cells (arrowhead) (H&E). (H) PHOX2B immunostaining highlights the rare mature‐appearing ganglion cells (arrowhead); preexisting adrenal gland tissue is seen in the lower left corner.

Gross findings and histological features are summarized in Figure 1.

### 3.2 Final Diagnosis

The histopathological diagnosis retained was a peripheral neuroblastic tumor NOS, with major spontaneous involutive fibrous changes and rare persistent mature ganglion cells without residual neuroblastic component, completely excised.

## 4 Outcome and Follow‐Up

The patient had an uneventful postoperative recovery. Postoperative clinical and ultrasound monitoring was recommended: every 6 months for the first 2 years, then annually for 5 years. No additional treatment was deemed necessary.

## 5 Discussion

Neuroblastic tumors are classified according to morphological criteria and spectrum of differentiation into neuroblastoma, ganglioneuroblastoma, and ganglioneuroma. Neuroblastoma is further divided into undifferentiated, poorly differentiated, and differentiated subtypes, depending on the presence or absence of neuropil and on the degree of neuroblastic differentiation. Intermixed and nodular ganglioneuroblastoma are described, whereas ganglioneuroma is subdivided into maturing and mature subtypes [10, 11, 12].

Post‐chemotherapy regression is well documented. It is typically characterized by a substantial reduction in tumor size (median decrease of 77.5%, range 60%–100%), and histologically associated with marked cellular loss, extensive fibrosis and calcification, moderate hemosiderin deposition, and occasional areas of necrosis [13].

However, although spontaneous regression without treatment has been reported in up to 50% of cases in infant localized neuroblastoma [7, 9], the histological features of neuroblastic tumor regression have been rarely reported in the literature, highlighting the need for this report.

Historical descriptions prior to 1990 describe the histological features of spontaneous regression in neuroblastoma, typically including necrosis, fibrosis, and calcification. In one case of stage 4S congenital neuroblastoma, with cutaneous and liver metastases, regression of the primary adrenal gland tumor was documented at 3 years of age; it remains unclear whether concurrent maturation into ganglioneuroma was present [14]. In another report, edema and necrosis were observed in a 0.5 cm in situ adrenal gland neuroblastoma arising within an otherwise normal‐appearing adrenal gland in a 1‐day‐old male infant. Neuroblast clusters were dispersed throughout the adrenal nodule, but these may represent residual developmental neuroblastic nests rather than true neoplastic elements [15]. Such changes could therefore reflect spontaneous involution of a lesion lacking neoplastic potential.

Several mechanisms have been proposed to explain both the development and regression of neuroblastoma. Brodeur and Bagatell [16] have summarized many of these in their review. One proposed factor is the expression of neurotrophin receptors, particularly TrkA, which plays a key role in neuroblastic differentiation. High TrkA expression has been observed in patients with low‐stage disease, and in vitro studies have shown that tumor cells expressing elevated levels of TrkA undergo apoptosis in the absence of nerve growth factor (NGF). Another hypothesis implicates immune‐mediated cell killing, potentially driven by anti‐neuroblastoma antibodies and natural killer (NK) cell activity, as a contributing factor to regression. Additionally, low telomerase activity and shortened telomeres have been associated with increased apoptosis and reduced tumorigenicity. In contrast, tumors with high telomerase activity often exhibit *MYCN* amplification, a well‐established marker of poor prognosis. Epigenetic mechanisms, including DNA methylation, histone modifications, and changes in chromatin remodeling, have also been implicated in neuroblastoma regression, as they can significantly influence gene expression and tumor behavior.

Most recently, pre‐cancerous spontaneous regression‐like features were observed in the Th‐MYCN mice model of neuroblastoma. Single‐cell transcriptomic analysis revealed a distinct ‘uncommitted’ neuroblastoma subtype, manifesting during early tumorigenesis, which was more abundant in samples from mice with suppressed tumor formation. These findings provide new insights into the mechanisms underlying spontaneous regression [17].

## 6 Conclusion

We have presented the histological features of a spontaneously regressing neonatal localized neuroblastoma, demonstrating clear similarities to post‐chemotherapy regression patterns. These findings may serve as a valuable reference for pathologists in recognizing and confirming spontaneous tumor regression during diagnostic evaluation.

## Author Contributions

**Tom Membrez:** writing – original draft. **Jim C. H. Wilde:** writing – review and editing. **Oliver Sanchez:** validation. **Danai Papangelopoulou:** writing – review and editing. **Fabienne Gumy‐Pause:** validation, writing – review and editing. **Anne‐Laure Rougemont:** data curation, supervision, writing – review and editing.

## Funding

The authors have nothing to report.

## Ethics Statement

We confirm that this report does not need to be reviewed by our ethics committee, the Geneva CCER, because the data of one case are not considered to be generalizable scientific results according to the Human Research Act (HRA). This Swiss Act applies to research concerning human diseases and concerning the structure and function of the human body.

## Consent

Written informed consent was obtained from the parents according to national and institutional guidelines.

## Conflicts of Interest

The authors declare no conflicts of interest.

## Data Availability

Data sharing is not applicable to this article as no datasets were generated or analyzed during the current study.
